# Ordered Porous TiO_2_@C Layer as an Electrocatalyst Support for Improved Stability in PEMFCs

**DOI:** 10.3390/nano11123462

**Published:** 2021-12-20

**Authors:** Gaoyang Liu, Zhaoyi Yang, Xindong Wang, Baizeng Fang

**Affiliations:** 1 Department of Metallurgical and Ecological Engineering, University of Science and Technology Beijing, 30 College Road, Beijing 100083, China; Zhaoyiyangustb@163.com (Z.Y.); echem@ustb.edu.cn (X.W.); 2 Beijing Key Laboratory for Magneto-Photoelectrical Composite and Interface Science, University of Science and Technology Beijing, 30 College Road, Beijing 100083, China; 3 Department of Chemical and Biological Engineering, University of British Columbia, 2360 East Mall, Vancouver, BC V6T 1Z3, Canada

**Keywords:** proton exchange membrane fuel cell, Pt-TiO_2_@C, ordered porous structure, electrochemical stability, electrocatalytic performance

## Abstract

Proton exchange membrane fuel cells (PEMFCs) are the most promising clean energy source in the 21st century. In order to achieve a high power density, electrocatalytic performance, and electrochemical stability, an ordered array structure membrane electrode is highly desired. In this paper, a new porous Pt-TiO_2_@C ordered integrated electrode was prepared and applied to the cathode of a PEMFC. The utilization of the TiO_2_@C support can significantly decrease the loss of catalyst caused by the oxidation of the carbon from the conventional carbon layer due to the strong interaction of TiO_2_ and C. Furthermore, the thin carbon layer coated on TiO_2_ provides the rich active sites for the Pt growth, and the ordered support and catalyst structure reduces the mass transport resistance and improves the stability of the electrode. Due to its unique structural characteristics, the ordered porous Pt-TiO_2_@C array structure shows an excellent catalytic activity and improved Pt utilization. In addition, the as-developed porous ordered structure exhibits superior stability after 3000 cycles of accelerated durability test, which reveals an electrochemical surface area decay of less than 30%, considerably lower than that (i.e., 80%) observed for the commercial Pt/C.

## 1. Introduction

Proton exchange membrane fuel cell (PEMFC) has been regarded as the main cell system for low/zero-emission electric vehicles and stationary energy applications in the future [1,2]. Among all kinds of catalysts, Pt has the highest catalytic activity in the cathode/anode reaction; however, its large use raises the cost of PEMFC [3,4,5]. Membrane electrode assembly (MEA), the core part of the PEMFC, is composed of gas diffusion layer (GDL), catalyst layer (CL), and proton exchange membrane [6]. In a traditional preparation process, the CL is sprayed on GDL, resulting in a loss of catalyst up to 50 percent, which has intensified the cost. Furthermore, the disordered pore channels between the support and catalyst enforce the water and gas to go through a long path, resulting in poor drainage and low Pt utilization [7]. Therefore, improving the stability and the utilization of Pt and building an ordered mass transport channel should be taken into consideration [8].

An ordered electrode structure can realize the separation of electrons, protons, and mass transport channels [9], which will reduce the polarization caused by the mass transport. In addition, it also increases the three-phase interface, reduces the required amount of Pt, and effectively improves the utilization of Pt catalyst during cell operation [10]. In this way, the power density and life of the fuel cell are significantly improved [11].

The ordered support structures commonly used in fuel cells mainly include the array structure of carbon [12], metal oxide [13], conductive polymer [14], and organic whisker represented by 3M company [15]. Pt nanoparticles are usually loaded on a support by magnetron sputtering [16], impregnation [17], hydrothermal [18], etc. The array with Pt nanoparticles can serve as an integrated cathode. However, some issues still remain during the practical operation of the fuel cell, including the agglomeration and the loss of the Pt nanoparticles [19,20,21]. This is mainly due to the instability of the carbon support [22,23]. The corrosion and oxidation of the support causes the loss of the supported Pt-based catalyst. Therefore, it is essential to provide a stable support for Pt-based catalysts [24].

Compared with the reported literatures, it is believed that both the improved microstructure and the interfacial composition between Pt nanoparticles and the support are extremely important towards the utilization and stability of Pt-based catalyst. In this work, a new integrated electrode with ordered porous core-shell structure is developed to afford fast electron transfer and mass transport, and therefore enhanced cycling stability. Titanium dioxide (TiO_2_), a low-cost and thermally stable semiconductor material [25,26], is utilized as the hollow porous core component of the support. Amorphous carbon is coated on the surface of TiO_2_ as the shell for improving the conductivity [27] and providing the active sites for Pt loading [28,29]. Due to the strong interaction of the support and Pt nanoparticles, stabilized electron, and mass transport, the integrated Pt-TiO_2_@C electrode structure has demonstrated considerably enhanced electrocatalytic activity and electrochemical stability compared with the commercial Pt/C and Pt nanowires catalysts.

## 2. Experimental Section

### 2.1. Materials

Hexachloroplatinic acid (H_2_PtCl_6_∙6H_2_O, 99.95%), formic acid (HCOOH, 88%), and other chemicals were purchased from Sinopharm Chemical Reagent Beijing Co., Ltd. (Beijing, China) Commercial 40% Pt/C (Johnson Matthey Co., London, UK), TGP-H-60 Carbon Paper (Toray, Tokyo, Japan), and all chemicals were used as received without any further purification.

**Figure 1:**
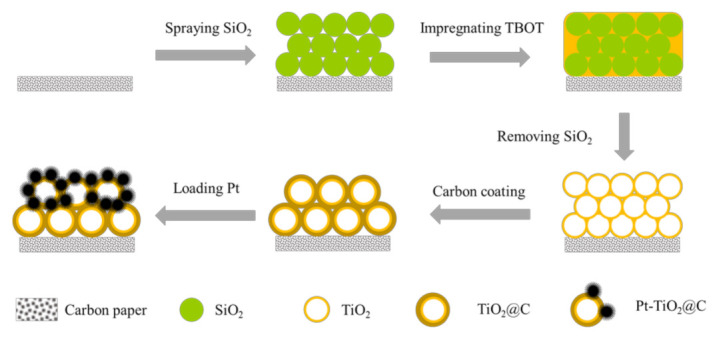
Schematic diagram for preparation of the porous Pt-TiO_2_@C array structure.

Figure 1 depicts the synthetic schematic diagram of the ordered porous array structure. First, the prepared uniform SiO_2_ particles are sprayed on the surface of the carbon paper (CP, Toray HCP030P) substrate. Then, the substrate is vertically placed into TiO_2_ precursor solution titanium (IV) butoxide (TBOT). Next, the silica template is removed by sodium hydroxide after drying and calcination. Later, a carbon layer is coated on the outside of the porous TiO_2_ by carbonization in Ar flow. Last, the porous TiO_2_@C is immersed in the chloroplatinic acid solution, and formic acid is added for the reduction of Pt(IV) and the growth of Pt nanoparticles at room temperature. The details about the synthesis of SiO_2_ sphere array, porous TiO_2_/CP array, porous TiO_2_@C/CP array, and Pt-porous TiO_2_@C/CP array can be seen in the following subsections [29].

### 2.2. Preparation Ordered Porous Pt-TiO_2_@C Array

#### 2.2.1. Preparation of SiO_2_ Sphere Array

SiO_2_ spheres were prepared by hydrolyzing the mixture solution containing a certain amount of tetraethoxysilane, deionized water, ethanol, and ammonia for 12 h at room temperature. Then, the product was collected by centrifugation at 10,000 rpm for 5 min, washed with ethanol and deionized water successively for several times, and dried at 60 °C overnight. A piece of CP, which was ultrasonically cleaned by acetone, deionized water, and alcohol in sequence, was used as the array substrate. SiO_2_ array was prepared by spraying SiO_2_ ink on the CP surface to form an ordered and uniform structure for subsequent experiments. The as-prepared SiO_2_ array is denoted as SiO_2_/CP.

#### 2.2.2. Preparation of Porous TiO_2_/CP Array

TiO_2_ precursor solution was prepared by the following steps. Ethanol and TBOT were mixed and stirred at a volume ratio of 14:1, and the prepared SiO_2_/CP was vertically immersed in the mixture solution for 5 min, then taken out and dried in an oven at 50 °C for 1 h. A TiO_2_ shell was formed outside the SiO_2_ after sintering under air atmosphere at 350 °C for 2 h. The sintered sample was placed in a 15% sodium hydroxide solution at 50 °C for 4 h to remove the silica template to obtain a porous ordered TiO_2_ hollow array structure.

#### 2.2.3. Preparation of Porous TiO_2_@C/CP Array

Carbon-coated porous TiO_2_ was obtained by a hydrothermal method. A total of 9.4 g of glucose was dissolved in 200 mL of deionized water [27]. A piece of porous TiO_2_/CP was placed at the bottom of the reaction kettle, and then 20 mL of the prepared glucose solution was poured into the kettle. The reaction was processed at 180 °C for 24 h. After cooling down to room temperature, the sample was dried at 80 °C followed by carbonization at 500 °C for 2 h in dry Ar flow. The sample was denoted as TiO_2_@C/CP.

#### 2.2.4. Preparation of Pt nanoparticle on Porous TiO_2_@C/CP Array

Pt nanoparticles anchored on the porous TiO_2_@C/CP array were prepared by a typical synthesis of chemical reduction at room temperature [28]. The porous TiO_2_ @C/CP substrate was immersed in aqueous solution of H_2_PtCl_6_ · 6H_2_O (50 mL, 5 mM) and HCOOH (88 wt%) (3 mL) to react for 72 h. After that, the product was washed and dried completely for further characterizations. The TiO_2_@C/CP support after platinum loading was used as an integrated cathode, and the amount of Pt was determined by accurately measuring the mass change before and after the Pt loading. The loading of Pt was measured to be 0.15 mg cm^−2^.

### 2.3. Structural Characterizations

The compositions of the as-synthesized samples were examined by X-ray diffraction (XRD, Rigaku RINT2400) and Raman spectroscopy (Jobin-Yvon, Bensheim, Germany). Scanning electron microscopy (SEM, JSM-7100F), transmission electron microscopy (TEM, FEI TecnaiF30), and energy-dispersive X-ray spectroscopy (EDX) were used to observe surface morphologies, microstructures, and elemental distributions. Chemical states of the samples were examined by X-ray photoelectron spectroscopy (XPS, Kratos AXIS ULTRA^DLD^).

### 2.4. Electrochemical Tests

Commercial 40wt% Pt/C was coated on the CP via a modified spray method reported by Liu et al. [30]. For comparison, Pt nanowires were grown on CP through the same method as shown in Section 2.2.4, and the platinum loadings for the two samples were also controlled to be ca. 0.15 mg cm^−2^. A piece of modified CP served as the working electrode (the geometric active area is 1.0 cm^2^). A saturated calomel electrode (SCE) served as the reference electrode, and a Pt plate was used as the counter electrode. Electrochemical tests were performed through a VMP2 (Bio-Logic, Seyssinet-Pariset, France) equipment at room temperature.

Electrochemical impedance spectroscopy (EIS) was used to evaluate the resistance of the samples in the frequency range of 0.01 Hz to 100 kHz at open circuit potential and 0.7 V in N_2_ and O_2_ saturated solution. Cyclic voltammetry (CV) curves were measured in 0.5M H_2_SO_4_ solution under a flow of N_2_ at a sweep rate of 50 mV s^−1^. Accelerated durability test (ADT) was performed in the potential range from 0.6 to 1.2 V (vs. reversible hydrogen electrode, RHE) for 3000 cycles [31], and the electrochemical surface area (ECSA) was evaluated by CV in the potential range from 0.05 to 0.4 V (vs. RHE) every 500 cycles by the following formula [32]:

$$S_{ECSA}\;=\;\frac{Q_{Integral\;area}}{2.1\;*\;50\;*\;m_{pt}}$$

## 3. Results and Discussion

**Figure 2:**
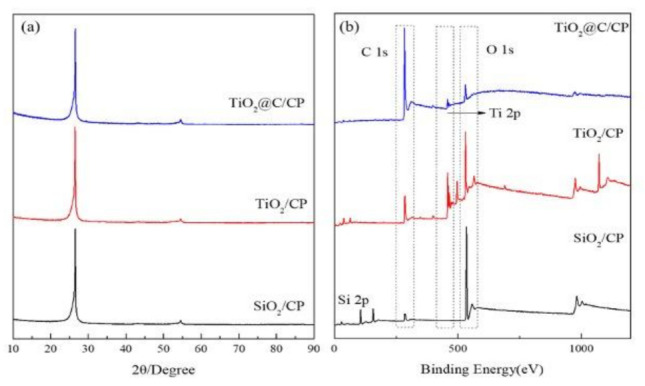
(**a**) XRD patterns and (**b**) XPS spectra of the SiO_2_, TiO_2_, and TiO_2_@C.

The XRD patterns before and after the impregnation of TiO_2_ are shown in Figure 2a. There are two diffraction peaks located at 26° and 54°, corresponding to graphite and turbostratic carbon in the CP, respectively. However, it is difficult to observe the corresponding diffraction peaks of SiO_2_, TiO_2_, and TiO_2_@C due to their low loadings on the CP support. Therefore, XPS measurements were used to evaluate the surface composition of the samples. As shown in Figure 2b, there is no obvious peak of Si 2p at the bind energy of 105.6 eV after removing the template, and only the peaks of Ti 2p were observed at the bind energy of 459.8 eV and 465.5 eV, which indicates that the silica template can be completely removed by sodium hydroxide solution. The strong peak at the binding energy of 529.2 eV corresponds to bulk oxygen bonded to titanium. After the carbon coating on the TiO_2_, the intensity of Ti 2p and O 1s peaks weakened. In contrast, the C 1s peak increased.

**Figure 3:**
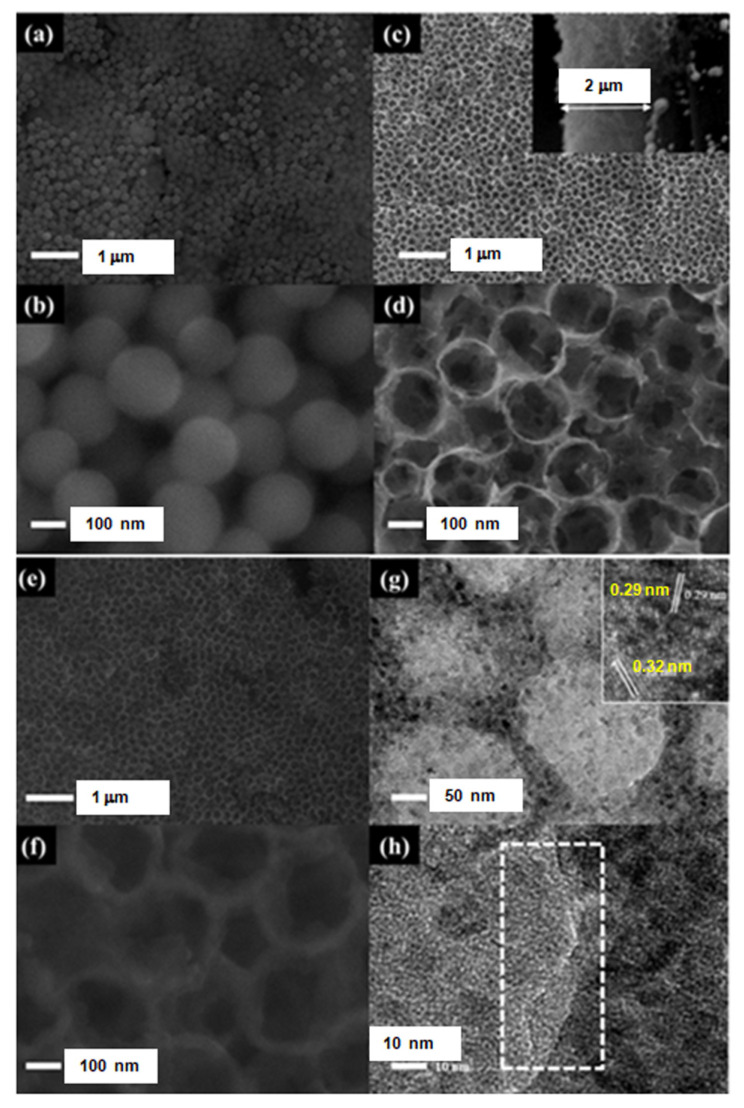
SEM images with various magnifications of SiO_2_ spheres modified CP (**a**,**b**), porous TiO_2_ modified CP (**c**,**d**), porous TiO_2_@C modified CP (**e**,**f**), and TEM images with various magnifications of TiO_2_@C (**g**,**h**).

Figure 3a,b show the SEM images of the ordered structure of SiO_2_. The average diameter of the prepared SiO_2_ is about 200 nm. After removing the SiO_2_ template, the ordered porous TiO_2_ array structure was obtained as shown in Figure 3c,d. It can be seen that the TiO_2_ precursor has completely infiltrated into the gap between the SiO_2_ spheres, and it converted into a porous hollow TiO_2_ structure after the removal of the SiO_2_ template. The porous hollow TiO_2_ has a diameter of about 200 nm, which is consistent with the SiO_2_ particle size and the thickness of the porous TiO_2_ layer is ca. 2 μm, as seen along the vertical direction in Figure 3c. For TiO_2_ support, Pt nanoparticles can be deposited on its surface by magnetron sputtering, UV reduction, and electrochemical deposition; however, it is difficult to grow the tiny and well-dispersed Pt nanoparticles by a simple approach. Interestingly, Pt is easy to grow on a carbon support. In order to provide sufficient active sites for homogeneous growth of Pt nanoparticles, a thin carbon layer was coated on the porous TiO_2_ to form the porous TiO_2_@C core-shell frame. As shown in Figure 3e, the ordered porous array does not change at low magnification compared to TiO_2_ array. Under the high magnification, as shown in Figure 3f, it is significantly different from Figure 3d, namely, the edge of the porous structure was thickened with the carbon layer. The core-shell structure and phase of the porous TiO_2_@C were further studied by TEM analysis. Figure 3g shows the porous TiO_2_@C core-shell structure with the carbon-coated layer, from which no significant change was observed in the morphology compared with the SEM images. In addition, in the part of core, TiO_2_ displays two kinds of lattice fringe spacing, 0.29 nm and 0.32 nm, corresponding to the diffraction fringe of the (001) and (110) facets of TiO_2_, respectively. Figure 3h reveals the high-resolution TEM of the porous TiO_2_@C, from which the carbon coated layer can be seen clearly. The amorphous carbon layer can be observed around the peripheral area, where there is no lattice fringe and the carbon layer is less than 10 nm in the thickness. This result is consistent with the XPS analysis. Evidently, porous TiO_2_@C core-shell nanostructure was successfully prepared, which was then used as the Pt catalyst support to form integrated cathode structure.

Pt was deposited on the TiO_2_@C/CP porous array at room temperature by formic acid reduction without capping agent. During the reaction, the solution changed from pale yellow to colorless, suggesting that the Pt(IV) species in the solution were completely converted to Pt(0). The reduction reaction took place as follows:

H_2_PtCl_6_ + 2HCOOH → Pt + 6Cl^−^ + 6H^+^ + 2CO_2_↑

**Figure 4:**
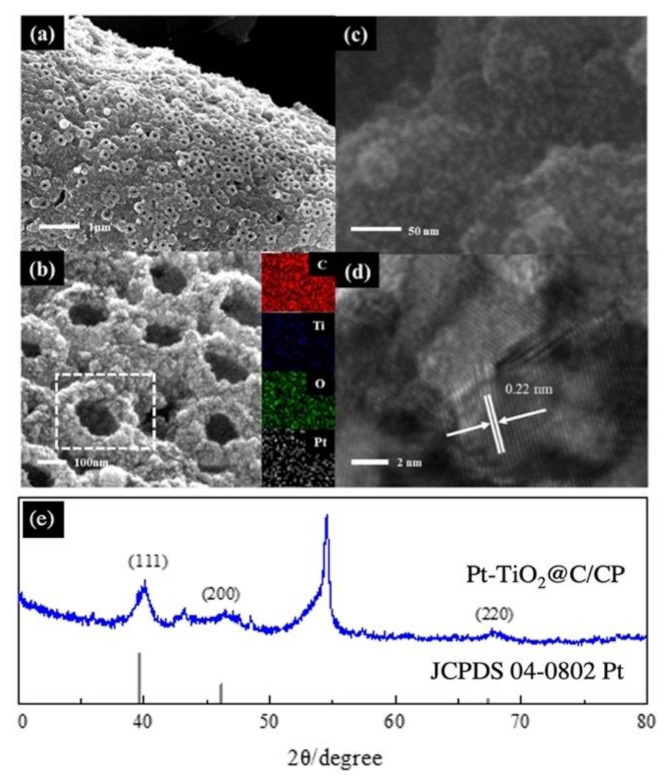
SEM (**a**–**c**) and TEM (**d**) images and XRD pattern (**e**) of the Pt-TiO_2_@C/CP.

Platinum tends to grow in the direction of (111) under the action of a weak reducing agent. Interestingly, the microstructure of Pt can be different with the surface composition of the substrate for the formic acid reduction of Pt. Pt nanowires were developed on the CP, similar to that reported previously for other substrates [29]. However, dense platinum nanoparticles were formed on the porous TiO_2_@C/CP, as seen in Figure 4. This may be due to the different preparation processes between the homemade and the traditional carbon layers. As seen from Figure 4a, Pt grows on the edge of the porous structure, and as a result, a few of the voids are covered by Pt and the rest of them remain in the form of pores. As shown in Figure 4b,c, at a high magnification, the structure of the porous Pt-TiO_2_@C/CP can be observed more clearly, and Pt presents in nanoparticles. The EDS element mapping was performed for the selected area in Figure 4b, which reveals a homogeneous distribution for C, Ti, O, and Pt on the surface of the sample. In Figure 4d, the lattice stripe of Pt can be clearly observed, and it is measured to be 0.22 nm, which is consistent with the (111) facet of XRD pattern in Figure 4e. The diffraction peak at ca. 39.6° can be assigned to the diffraction at Pt (111). According to Scherrer Equation, the average crystalline size of Pt nanoparticles is calculated to be ca. 4.9 nm for the Pt-TiO_2_@C/CP, which is significantly larger than the size (ca. 2.9 nm) of the Pt nanoparticles observed for the commercial 40wt% Pt/C (JM) in our previous study [2].

**Figure 5:**
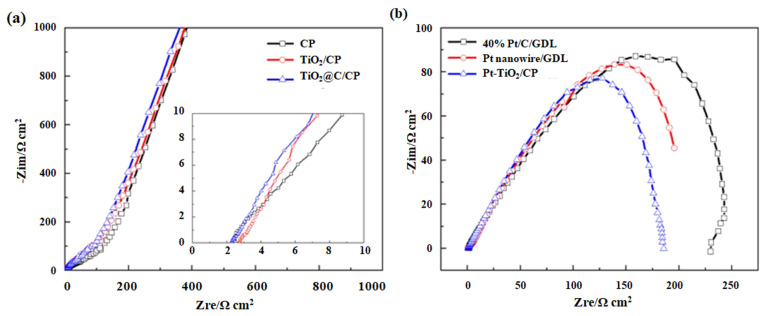
EIS plots of the CP, TiO_2_/CP, and TiO_2_@C/CP (**a**) and Pt/C, Pt nanowire/GDL, and Pt-TiO_2_@C/CP (**b**).

Figure 5a shows the Nyquist plots for the various catalyst supports and various supported Pt catalysts. As a semiconductor material, the conductivity of TiO_2_ is significantly lower than ordinary CP, and as a result, a higher charge transfer resistance was observed for this material (i.e., TiO_2_/CP). After the carbon coating, the electrochemical impedance is significantly reduced because the presence of the carbon layer increases the conductivity. The resistance values of the CP, TiO_2_/CP and TiO_2_@C/CP are 2.52, 2.75, and 2.29 Ω cm^2^, respectively. The presence of the carbon layer not only reduces the electrical resistance but also provides abundant sites for Pt nanoparticle growth, as described previously. Figure 5b compares the electrochemical impedances at 0.7 V for the various catalysts. From the Nyquist curves, the resistance values are measured to be 225, 200, and 180 Ω cm^2^ for Pt/C/GDL, Pt nanowire/GDL, and Pt-TiO_2_@C/CP, respectively. The commercial Pt/C shows the biggest impedance, but it is significantly decreased by optimizing the morphology of Pt or the synergistic effect between the metals. Since the electrochemical data were not recorded in a single cell, the electrochemical impedance is large.

**Figure 6:**
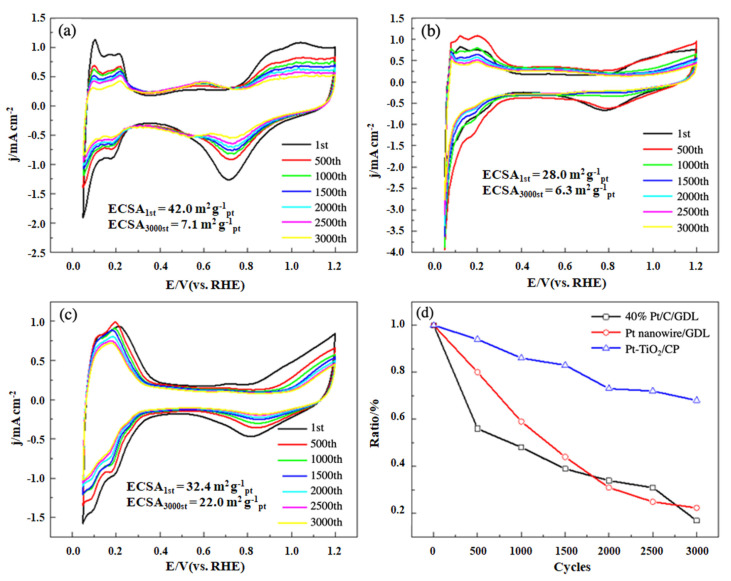
CV curves of conventional Pt/C [33], Pt nanowire, and Pt-TiO_2_@C ordered cathode before and after 3000 ADT cycles (**a**–**c**) and the decay of ECSA (**d**).

Figure 6a–c show the CV curves of the commercial Pt/C, Pt nanowire, and Pt-TiO_2_@C integrated electrodes. The ECSAs were obtained by integrating from 0.05 V to 0.4 V after deducting the double layer. Through the calculations, the ECSA of the commercial MEA cathode is 42.0 m^2^ g^−1^_Pt_, while the ECSA of the Pt nanowire is only 28.0 m^2^ g^−1^_Pt_, and it is 32.4 m^2^ g^−1^_Pt_ for the Pt-TiO_2_@C integrated catalyst cathode. The ECSAs of the latter two catalysts are smaller than the commercial one. This is because the surface area of the nanowire-shaped Pt is significantly lower than that of the Pt nanoparticle, consistent with the results reported for the Pt nanowires by other scholars [32]. Although Pt exists in the form of nanoparticles in the porous Pt-TiO_2_@C array structure, the lower active area may be due to the fact that the surface area of the metal oxide-based TiO_2_ support is smaller than that of the carbon-based support, according to our previous studies [26,29]. During the ADT, the ESCAs reduced for all the tested catalysts, but the decay in the active area of the integrated electrode is slow. The curves of ECSA decay are displayed in Figure 6d. After 3000 cycles of the ADT, only 20% ECSA remained for the pure carbon powder catalyst (i.e., commercial Pt/C). Especially during the first 500 cycles, a more significant drop in the ECSA was observed for this catalyst. Interestingly, the Pt-TiO_2_@C shows a much higher electrochemical stability, and up to 70% of the ECSA is still maintained after 3000 cycles of ADT.

**Figure 7:**
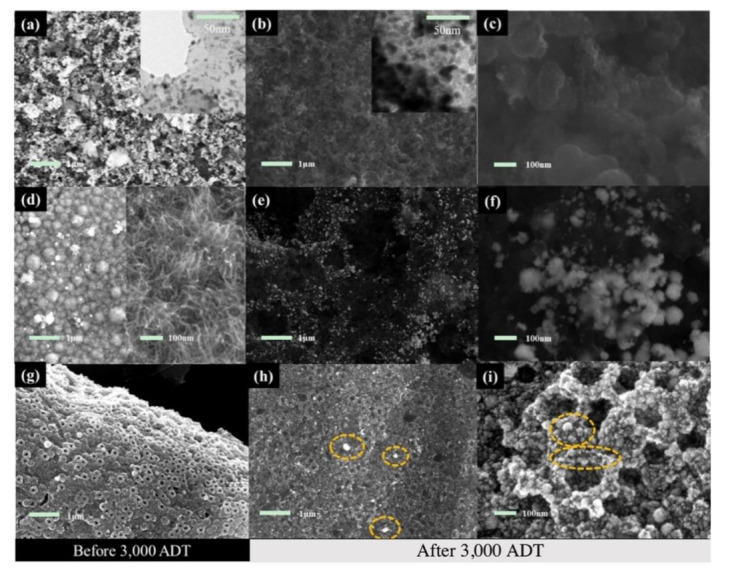
SEM and TEM images of Pt/C/GDL cathode (**a**–**c**), Pt nanowire/GDL cathode (**d**–**f**), and Pt-TiO_2_@C porous ordered cathode (**g**–**i**) before and after 3000 cycles of the ADT.

The improved electrochemical stability is mainly due to the fact that the platinum nanoparticles do not significantly aggregate and fall off on a nonpure carbon support during the prolonged testing, which can be seen from the SEM images shown in Figure 7. After the accelerated test, the commercial catalyst had accumulated nanoparticles and thus reduced the utilization of platinum, as shown in Figure 7a–c. A similar phenomenon was also observed for the Pt nanowires on carbon support from the images shown in Figure 7d–f. Interestingly, the Pt nanoparticles on the porous TiO_2_@C remained almost unchanged after the test, indicating the porous TiO_2_@C has better stability than conventional carbon layers. As shown in Figure 7h, the white spots in the circles are the aggregated Pt nanoparticles, and, due to the accumulation of Pt nanoparticles, it is inevitable that some parts of the catalyst support have no Pt loading and the surface is directly exposed. The naked supports (i.e., vacant sites) can be seen from the image shown in Figure 7i and are present around the accumulated Pt spheres. This explains why the ECSA of the Pt-TiO_2_@C decayed after 3000 cycles of the ADT.

In the meantime, another important finding was that the oxygen reduction peak potential recorded on the Pt nanowire or Pt-TiO_2_@C moves in the positive direction. The CV curves revealed an oxygen reduction peak potential of 0.71 V for the Pt/C, 0.79 V for the Pt nanowire, and 0.82 V for the Pt-TiO_2_@C, as shown in Figure 6. The positive shift of the peak potential indicates that, at a certain potential, it has a higher specific oxygen reduction reaction activity with the equivalent Pt loading. The first positive shift of the oxygen reduction peak is due to the special structure of the nanowires that enables Pt to release more active sites or have less defects, as mentioned in literature [32]. The further forward shift of the oxygen reduction peak may be attributed to the strong attraction between Pt and TiO_2_, and the synergistic effect between them increases the catalytic activity.

**Figure 8:**
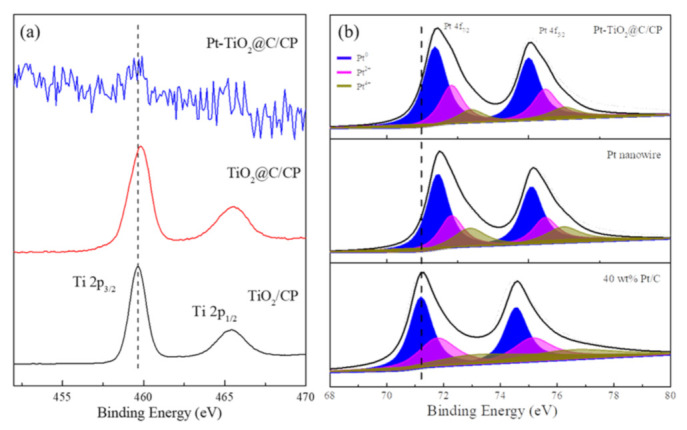
XPS narrow spectra of Ti 2p (**a**) and Pt 4f (**b**) in different samples.

XPS analysis is a useful tool to further identify the specific reasons for the improvement in the catalytic activity and electrochemical stability. Figure 8a shows the XPS spectra of the TiO_2_, TiO_2_@C, and the integrated Pt-TiO_2_@C electrode. The element of Ti 2p in TiO_2_ at the binding energy of 459.7 eV and 465.4 eV correspond to the Ti 2p_1/2_ and Ti 2p_3/2_ peaks, respectively. After the carbon layer coating, the binding energy moves in a positive direction, probably due to the formation of highly oxidized Ti-O bonds [33]. After the Pt was deposited on the surface of the TiO_2_@C, the peak positions of the Ti remain almost unchanged compared to TiO_2_, but display a negative shift compared with the TiO_2_@C/CP, indicating that the interaction between TiO_2_ and Pt is formed. A pair of Pt XPS peaks can be seen in Figure 8b, which proves the successful loading of Pt onto the surface of the support. The commercial Pt/C shows the peaks at 71.2 eV and 74.6 eV for Pt 4f_5/2_ and Pt 4f_7/2_, respectively, while the electron binding energy of the Pt nanowire are 71.8 eV and 75.0 eV, which reflects the shift of the peak potential to a high binding energy. This result is partly attributed to the special structure of the platinum nanowire. For the Pt-TiO_2_@C, the Pt peaks also move towards a high binding energy, with the binding energies of 71.7 eV and 74.9 eV for Pt 4f_5/2_ and Pt 4f_7/2_, respectively. The combined energy transfer results from the weak attraction between titanium and platinum, as mentioned before. In addition, it can be seen from the peaks that most of the Pt exists in a zero-valence state, and the rest exists in the form of divalent and tetravalent Pt. The area ratio can be used to confirm the relative content of Pt with different chemical valences. The ratio values of the three samples are 2.7:1.4:1.0 (Pt^0^:Pt^2+^:Pt^4+^), 3.3:1.4:1.0, and 5.4:2.5:1.0, respectively. Evidently, most of the Pt prepared by this immersion reduction method exists in a zero-valence state. Hence, more active Pt can be used for the electrochemical catalytic reaction.

**Figure 9:**
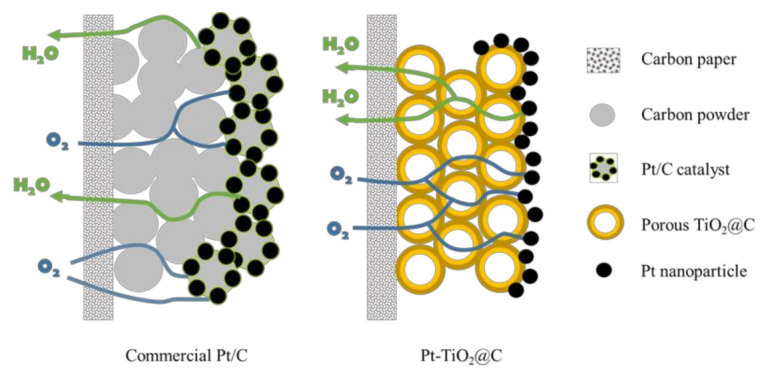
Schematic diagram of mass transfer of Pt/C and Pt-TiO_2_@C ordered array structure.

In order to clearly highlight the importance of the ordered array structure in the as-developed Pt-TiO_2_@C catalyst, a schematic diagram is displayed in Figure 9, which illustrates the water and vapor transport in the carbon powder layer and porous TiO_2_@C layer. In a disordered carbon powder layer structure, the reactants/products will transport through a longer path, while the electrons need to be transported through a continuous conductive material, and ions transport is also performed in a disordered state way. Each of the mass transfers is in a state of chaos, which is not conducive to the rapid progress of the electrode reaction. As for the porous TiO_2_@C support, both gas and liquid can be transported through the porous TiO_2_@C. The porous electrode structure is more beneficial for the rapid mass transfer, and the electrons will transport through the TiO_2_@C to reach the Pt nanoparticle surface. Furthermore, for the commercial Pt/C, after a long period of operation, some channels lose the mass transfer ability because of the carbon support corrosion and the aggregation or even detachment of Pt nanoparticles. As shown in Figure 7, the mass transfer channels are diminished, and the mass transfer resistance increases as a result. In contrast, for a stable porous ordered structure, the mass transfer channel does not change significantly after a long period of operation, as confirmed from the SEM observation in Figure 7h. In general, the ordered structure has better mass transfer capability and superior stability. Moreover, the unique core-shell structure composed of a hollow macroporous core connected with the porous shell in the TiO_2_@C affords fast mass transport and enhanced cycling stability. Such important impacts have been frequently reported in literature for diverse catalysis and electrochemical energy-related applications [34,35,36,37,38,39,40,41,42,43,44,45].

## 4. Conclusions

Pt nanoparticles were successfully loaded onto the porous TiO_2_@C ordered array by immersion reduction. The TEM observations prove the core-shell structure of the TiO_2_@C, while the SEM and XPS analyses reveal that Pt is more likely to grow into nanoparticles on the surface of the carbon layer. Compared with the conventional Pt/C and Pt nanowire catalysts, the ordered porous support structure could accelerate the mass transport inside the electrode, confirmed by the significantly reduced impedance recorded at 0.7 V vs. RHE. In addition, the array structure enhanced the stability of the support. After 3000 cycles of the ADT, the decay of ECSA was less than 30%, which provided a guarantee for the long-term stable operation of the fuel cell. Hence, this study is believed to provide an innovative strategy for the synthesis of an ordered porous array structure to prepare stable membrane electrode with high Pt utilization for PEMFC and other electrochemical applications.

## Data Availability

The study did not report any data.
